# Alpha-peptide receptor radionuclide therapy using actinium-225 labeled somatostatin receptor agonists and antagonists

**DOI:** 10.3389/fmed.2022.1034315

**Published:** 2022-12-07

**Authors:** Mengqi Shi, Vivianne Jakobsson, Lukas Greifenstein, Pek-Lan Khong, Xiaoyuan Chen, Richard P. Baum, Jingjing Zhang

**Affiliations:** ^1^Department of Diagnostic Radiology, Yong Loo Lin School of Medicine, National University of Singapore, Singapore, Singapore; ^2^Nanomedicine Translational Research Program, NUS Center for Nanomedicine, Yong Loo Lin School of Medicine, National University of Singapore, Singapore, Singapore; ^3^Academy for Precision Oncology, International Centers for Precision Oncology (ICPO), Wiesbaden, Germany; ^4^CURANOSTICUM Wiesbaden-Frankfurt, Center for Advanced Radiomolecular Precision Oncology, Wiesbaden, Germany; ^5^Clinical Imaging Research Centre, Centre for Translational Medicine, Yong Loo Lin School of Medicine, National University of Singapore, Singapore, Singapore; ^6^Department of Surgery, Chemical and Biomolecular Engineering, and Biomedical Engineering, Yong Loo Lin School of Medicine and College of Design and Engineering, National University of Singapore, Singapore, Singapore; ^7^Agency for Science, Technology, and Research (A*STAR), Institute of Molecular and Cell Biology, Singapore, Singapore

**Keywords:** actinium-225, neuroendocrine tumor, peptide receptor radionuclide therapy (PRRT), targeted α-particle therapy, SSTR, SSTR antagonist

## Abstract

Peptide receptor radionuclide therapy (PRRT) has over the last two decades emerged as a very promising approach to treat neuroendocrine tumors (NETs) with rapidly expanding clinical applications. By chelating a radiometal to a somatostatin receptor (SSTR) ligand, radiation can be delivered to cancer cells with high precision. Unlike conventional external beam radiotherapy, PRRT utilizes primarily β or α radiation derived from nuclear decay, which causes damage to cancer cells in the immediate proximity by irreversible direct or indirect ionization of the cells’ DNA, which induces apoptosis. In addition, to avoid damage to surrounding normal cells, PRRT privileges the use of radionuclides that have little penetrating and more energetic (and thus more ionizing) radiations. To date, the most frequently radioisotopes are β^–^ emitters, particularly Yttrium-90 (^90^Y) and Lutetium-177 (^177^Lu), labeled SSTR agonists. Current development of SSTR-targeting is triggering the shift from using SSTR agonists to antagonists for PRRT. Furthermore, targeted α-particle therapy (TAT), has attracted special attention for the treatment of tumors and offers an improved therapeutic option for patients resistant to conventional treatments or even beta-irradiation treatment. Due to its short range and high linear energy transfer (LET), α-particles significantly damage the targeted cancer cells while causing minimal cytotoxicity toward surrounding normal tissue. Actinium-225 (^225^Ac) has been developed into potent targeting drug constructs including somatostatin-receptor-based radiopharmaceuticals and is in early clinical use against multiple neuroendocrine tumor types. In this article, we give a review of preclinical and clinical applications of ^225^Ac-PRRT in NETs, discuss the strengths and challenges of ^225^Ac complexes being used in PRRT; and envision the prospect of ^225^Ac-PRRT as a future alternative in the treatment of NETs.

## Introduction

Neuroendocrine tumors (NETs) are well-differentiated, low proliferating neuroendocrine neoplasms (NENs) (1), most commonly arising from gastroenteropancreatic structures and the lung, although NEN have been described in almost every tissue. Accounting for only 0.5% of all malignancies, NETs are considered rare (2), however, the incidence/prevalence has been increasing in many epidemiological studies over the last decades (with GEP NETs demonstrating the highest incidence rate with 3.56 cases per 100,000) (2–7). The WHO grading system relies extensively on the proliferation rate to classify low proliferative NETs (NET-G1) with good prognosis, intermediate grade (NET-G2), and high grade (NET-G3) that show poor prognosis (8).

As a heterogeneous disease with very diverse symptomatology, NETs require multidisciplinary treatment and care, including medical control, surgery, chemotherapy, and internal or external radiation therapy (9). The cornerstone of therapy is still surgery with curative intent, whenever possible. However, in the case of metastatic disease, total excision is generally not possible due to the infiltration of other tissues and/or blood vessels or the number of metastatic sites (10, 11).

Systemic chemotherapy provides only modest benefit in rapidly proliferating tumors (grade 3) (12, 13). Therapeutic options such as somatostatin analogs (SSAs) or interferon-α may improve symptoms caused by hormonal excess or even lengthen the time to disease progression by offering hormonal and antiproliferative control over NETs, but rarely lead to partial or complete tumor response (14, 15). External beam radiotherapy (EBRT) unfortunately is not effective for the treatment of metastasized and secondary cancer sites beyond the treatment area (16, 17).

Theranostics, the concept of combining the inevitably intertwined arts of diagnostics and therapy, is a treatment option that has gained momentum over the last two and a half decades. Peptide receptor imaging and peptide receptor radionuclide therapy (PRRT) were the first successful examples of the theranostic concept, for imaging and treating cancer. PRRT has long been considered as a palliative treatment for NETs, but is now attracting more and more attention as a very effective symptomatic and well-tolerated treatment prolonging progression-free (and possibly overall) survival. As a complement to surgery, neoadjuvant therapy can make previously difficult-to-operate tumors operable by shrinking them, and as an adjuvant therapy, it may prevent tumor re-growth after surgical manipulation and growth of pre-existing micrometastases (18, 19).

Unlike chemotherapy and EBRT, PRRT targets disease at the cellular level in the systemic treatment of non-resectable and metastasized NETs (16). The overexpression of somatostatin receptors (SSTRs) of various sub-types in about 80% of NETs provides a continuously evolving way to diagnose and treat NETs (18, 20). The working principle of PRRT is using a therapeutic radionuclide chelated to a SSTR binding peptide and as the compound binds to SSTR expressing tissue, DNA-damaging radiation is delivered nearly exclusively to tumor cells and its microenvironment while sparing the surrounding healthy tissue. Somatostatin, the native peptide, is an obvious example of SSTR-binding peptide (21). However, it is susceptible to fast enzymatic degradation and is thus not suitable for *in vivo* applications (22). Instead, synthetic peptides, including those based on SSAs, have been developed with the intent to optimize metabolic stability, tumor retention time, and affinity.

## History of peptide receptor radionuclide therapy

**FIGURE 1:**
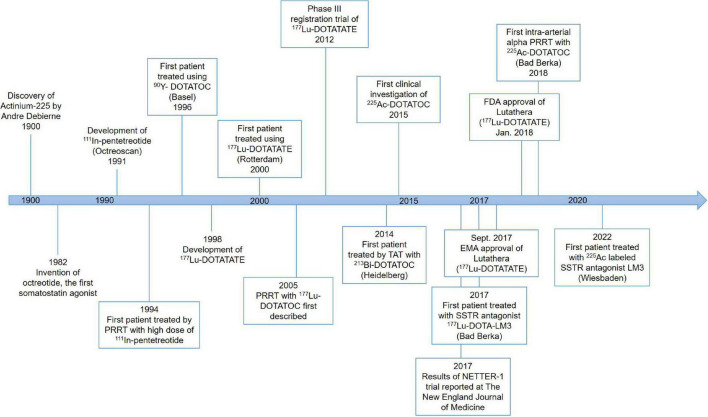
History of somatostatin-based peptide receptor radionuclide therapy (PRRT).

The first PRRT was performed in the early 1990s (Figure 1). The Rotterdam group successfully developed [^111^In-DTPA-_D_-Phe^1^]-octreotide (^111^In-pentetreotide) somatostatin scintigraphy (Octreoscan), and subsequently examined its imaging potential in more than 1,000 patients (23–25). Based on high uptake of ^111^In-pentetreotide by tumors as demonstrated by imaging, Krenning’s team successfully treated a patient with metastatic glucagonoma using a high dose of ^111^In-pentetreotide, which resulted in a decreased level of circulating glucagon as well as decreased tumor size (26).

This early work set the stage for further development of this exciting new field of radiomolecular precision medicine. For example, guided by the experience with PRRT using ^111^In-pentetreotide, the need for more suitable radionuclides was identified because the properties of ^111^In (decay by electron capture with a half-life of 2.8 days) do not provide good tissue penetration which corresponds with a modest or no tumor shrinkage. Improvement of PRRT has been made tremendously since then due to the development and availability of novel peptides, chelators, and radionuclides in various combinations (27).

**FIGURE 2:**
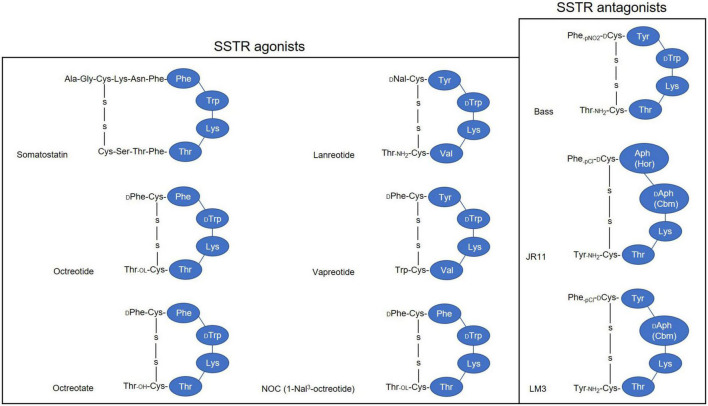
Simplified illustration of somatostatin receptor agonists and antagonists. 1-Nal, naphthyl-alanine; Aph(Hor), 4-amino-L-hydroorotyl -phenylalanine; D-Aph(Cbm), D-4-amino-carbamoyl-phenylalanine.

Derivatizing [Tyr^3^]-octreotide (Figure 2), which has a higher binding affinity for SSTR2 than the natural somatostatin analog (SSA) octreotide, and combining it with the chelator 1,4,7,10-tetra-azacyclododecane-tetra-acetic acid (DOTA) enabled stable radiolabeling with the high-energy beta particle-emitter Yttrium-90 (^90^Y-DOTATOC).

This therapeutic moiety was first applied in a pilot study for the treatment of three patients with abdominal metastases of neuroendocrine carcinoma of unknown localization (28, 29); and its therapeutic potential was evaluated subsequently with larger SSTR-positive patient numbers (30, 31). Treatment with ^90^Y-DOTATOC stopped rapid tumor progression, decreased the tumor marker neuron-specific enolase (NSE), and allowed disease stabilization (28, 30, 31). DOTATOC has since become a popular theranostics agent, demonstrating superior diagnostic sensitivity compared to Octreoscan, and demonstrating promising therapeutic value for treating SSTR-positive NETs when labeled with β- emitters, particularly Yttrium-90 (^90^Y) and Lutetium-177 (^177^Lu).

Another extensively studied SSA is DOTA-[Tyr^3^]-octreotate (DOTATATE), where the alcohol Thr(ol) of the C-terminus of DOTATOC is replaced by the natural amino acid Thr. This somatostatin analog was developed in 1998 and was found to show an even higher affinity to SSTR2 and a higher uptake in pancreatic tumor cells compared to the previously described SSAs (32).

The first clinical trial with ^177^Lu-DOTATATE started in 2000 in Rotterdam, The Netherlands, and led to the multinational phase three trial named NETTER-1 (33, 34). In this randomized controlled trial, a significantly higher response rate and extended progression-free survival were demonstrated in patients with advanced progressive SSTR-positive midgut NETs, compared to the double dose of long-acting repeatable (LAR 60 mg) octreotide administrations (18, 27, 34, 35). In January 2018, ^177^Lu-DOTATATE under the name of Lutathera was approved by the Food and Drug Administration (FDA) for the treatment of SSTR2-positive gastroenteropancreatic NETs (GEP-NETs) in adults (36). The approval of Lutathera in Europe was granted by the European Medicines Agency (EMA) already in September 2017 (37).

Other well-known somatostatin SSAs include lanreotide and vapreotide, but as these are not approved for PRRT of NETs, possibly due to their affinity pattern to SSTR subtypes and mode of action (38–40), they will not be discussed in this review.

Despite the success of [^90^Y]Y-DOTATOC and [^177^Lu]Lu-DOTATATE in the treatment of NETs in terms of progression-free survival, there were problems with ^90^Y concerning renal toxicity and a low rate of complete remissions, suggesting an improvement in PRRT efficacy is required (27, 41) with the main aspects of (1) identification of prognostic and predictive factors; (2) α-PRRT using ^225^Ac labeled ligands; and (3) shift from using SSAs to somatostatin antagonists.

## ^68^Ga-labeled somatostatin analogs for imaging (somatostatin receptor-PET)

Even with the success of DOTATOC and DOTATATE, clinicians need to take the individual variability in the therapy response of patients with seemingly similar profiles into account. ^68^Ga is a positron emitter that can be chelated to DOTATOC or DOTATATE for PET/CT imaging before therapy. SSTR-PET/CT with ^68^Ga-labeled DOTATATE and/or DOTATOC showed very promising results (42). All studies agreed on the important role of SSTR-PET using ^68^Ga for NET imaging and therapy planning, supporting the potent theranostic role of a radiolabeled DOTA-peptide (43–48). As a result, ^68^Ga-DOTATOC was approved by the FDA in 2019 as the first ^68^Ga-labeled radiopharmaceutical for imaging of SSTR positive GEP-NET using PET (49).

## Choosing a radionuclide for peptide receptor radionuclide therapy

Table 1 lists the physical properties regarding the clinically most frequently used radioisotopes in PRRT of NETs.

**TABLE 1:** Physical properties of selected radioisotopes used in PRRT to treat NETs.

Radionuclide	Decay	Half-life/h	Energy (max)/keV	Tissue penetration depth/mm	Application
^111^In	EC, γ	67.2	245 19	0.5	Imaging
^90^Y	β^–^, γ	64.2	2,284	11	Therapy
^177^Lu	β^–^, γ	160.8	498	1.7	Imaging/therapy
^68^Ga	β^+^	1.13	1,920		Imaging

EC, electron capture.

While ^68^Ga is useful for imaging purposes, all other radionuclides in Table 1 are used both for therapy and for single-photon emission computed tomography (SPECT) imaging. Out of these, ^90^Y and ^177^Lu are the two favorable isotopes due to their higher particle energies compared to ^111^In. ^90^Y (*t*_1/2_ = 64.2 h) emits β^–^-particles with a maximum energy of 2.284 MeV, allowing penetration of soft tissue to a depth of around 11 mm (50, 51). Because of its longer range compared to ^177^Lu, ^90^Y-DOTATE/TOC is suggested to be more suitable for bigger lesions, while ^177^Lu-DOTATATE might be preferred for smaller lesions (52). It has been demonstrated that ^90^Y-PRRT has a small potential of causing renal toxicity when used without nephroprotection, especially in patients with compromised renal function (53, 54). For ^177^Lu-DOTATATE or ^177^Lu-DOTATOC no significant renal damage occurred even in long-term follow up studies. An increasing number of clinical studies suggest that the combination of ^90^Y and ^177^Lu could be better than either radionuclide alone for PRRT in NETs, with an improved overall survival (55, 56). In parallel to β^–^-emitters, radionuclides emitting α-particles recently are gaining special interest for the treatment of NETs.

## Why alpha in peptide receptor radionuclide therapy

To date, most PRRTs rely on β-emitters, especially ^90^Y and ^177^Lu, because of the availability of these radioisotopes and the proven clinical effect. However, due to the relatively large range of these radionuclides surrounding normal tissues are also exposed to radioactivity. Furthermore, hypoxic cancer tissue could be resistant to β-emitter treatment, causing radiotherapeutic failure of β-PRRT (57, 58).

**FIGURE 3:**
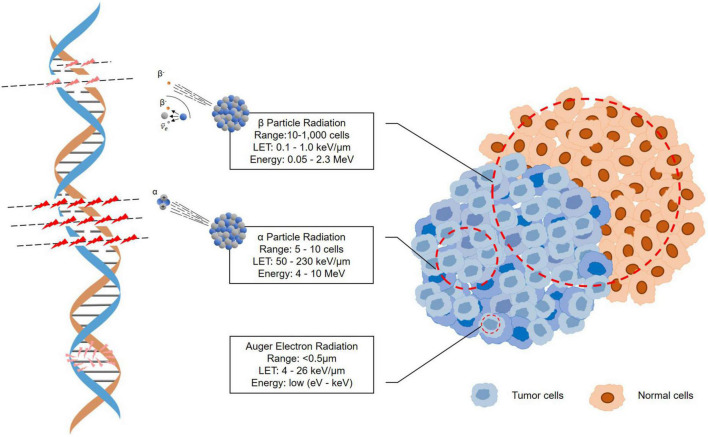
Illustration of the tracks of α-particle, β-particle, and auger election radiation.

Targeted α-particle therapy (TAT) offers a therapeutic option for patients resistant to β-irradiation treatments. An α-particle is a helium-4 (^4^He) nucleus consisting of two protons and two neutrons with an overall charge of +2 (59). Because of the double-positive charge, α-particles deliver dense ionization along a linear track, often described as high linear transfer (LET), ranging from 50 to 230 keV/μm (Figure 3) (60). This high LET renders higher target cell toxicity originating from higher probability of DNA double strand breaks (DSB) compared to β-particles with low LET (0.1–1.0 keV/μm) (61). Moreover, the primary target of high-LET α-particle is DNA, and a low number of particles can result in irreparable DSBs and lack of oxygen effects on cytotoxicity (62–64). Thus, the cytotoxicity of α-particle may be extremely effective and may also be more dose independent than β-emissions with cell death occurring from a single or a few α-particle traversals of the cell nucleus (65, 66). On the other hand, the typical tissue range of α-particles does not exceed 100 μm, which is significantly shorter than that of β-particles (0.05–12 mm) (Figure 3) (16, 58). This allows for selective ablation of the targeted tumor cells whilst minimizing the damage to surrounding healthy tissues (67).

## Why actinium-225

Considering the half-life, production, availability, and ability to be stably incorporated into a suitable vector, only a handful α-radionuclides have potential for clinical use, including actinium-225, bismuth-213, astatin-211, thorium-227, radium-223, radium-224, lead-212, bismuth-212, and terbium-149 (Table 2) (57, 64, 68–75).

**TABLE 2:** Medically relevant α-emitters with their decay properties.

Parent	Daughter	*t*_1/2_	α -decay	α -energy/MeV	Emissions per decay	Radiolabeling approach
^225^Ac		9.9 days	100%	5.94	4α, 3β^–^	Chelation by DOTA or NETA
	^213^Bi	46 min	2.2%	5.87	1α, 2β^–^	Chelation by DTPA or DOTA
^211^At		7.2 h	42%	5.87	1α, 1EC	Radioastatination
^227^Th		18.7 days	100%	6.14	5α, 2β^–^	Chelation by DOTA
	^223^Ra	11.4 days	100%	5.71	4α, 2β^–^	
^224^Ra		3.6 days	100%	5.69	5α, 2β^–^	
	^212^Pb	10.6 h		6.09	1α, 2β^–^	Chelation by TCMC
	^212^Bi	1.0 h	36%	6.05	1α, 1β^–^	Chelation by DTPA or DOTA
^149^Tb		4.1 h	17%	3.96	1α, β^+^	

α-emitters that are considered not suitable for therapeutic use are not listed. EC, electron capture.

**FIGURE 4:**
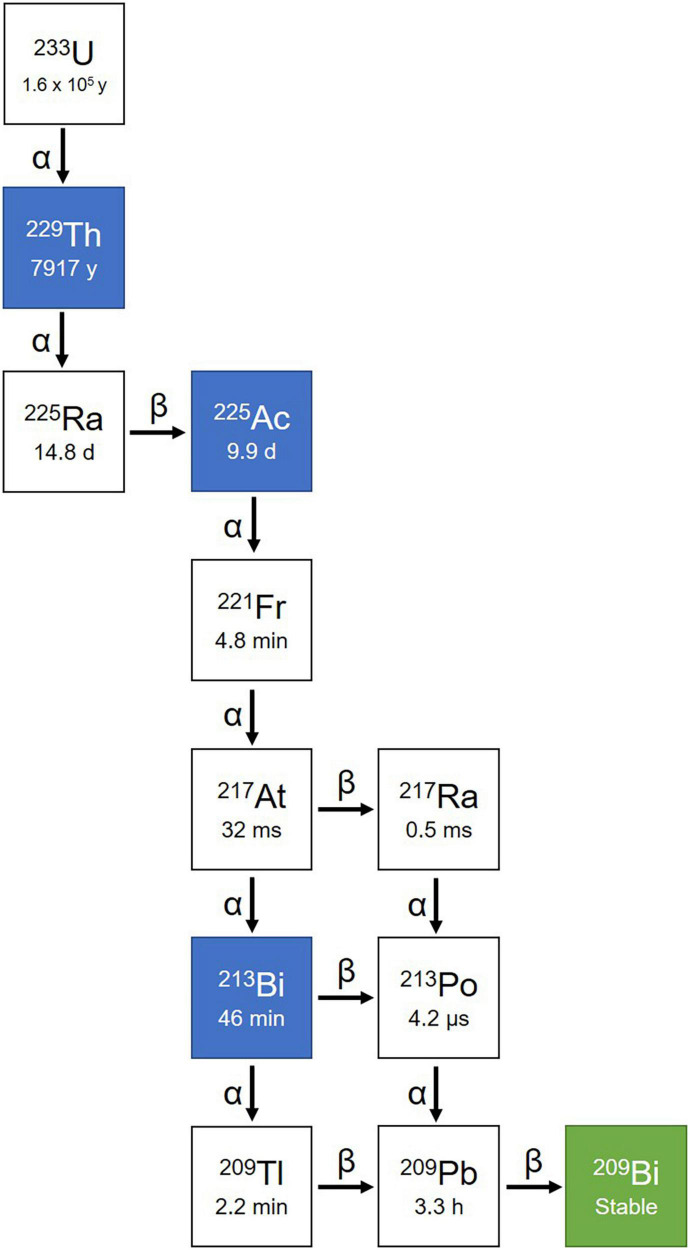
Decay chain of ^225^Ac. ^225^Ac decays to ^209^Bi with seven intermediate radionuclide progenies, including ^221^Fr, ^217^At, ^213^Bi, ^209^Tl, ^217^Ra, ^213^Po, and ^209^Pb. Among the decay chain ^225^Ac and ^213^Bi are medically relevant and intensively investigated.

Among all medically relevant α-particles, the generator derived radionuclide ^225^Ac (and its daughter radionuclide ^213^Bi) are considered particularly promising. ^225^Ac was discovered by Andre Debierne in 1899 and Friedrich Giesel in 1902 (76). As a pure α-emitter with a half-life of 9.9 days, the decay of ^225^Ac produces seven radionuclide daughters in the decay chain to stable ^209^Bi (Figure 4). From this decay path, a single ^225^Ac decay yields a total of four α, three β^–^ disintegrations, and two γ emissions. As such, ^225^Ac is classified as nanogenerator or *in vivo* generator (77). The relatively long half-life, the multiple α-particle emissions in the decay chain, and the rapid decay to stable ^209^Bi makes ^225^Ac a candidate of great potential for application in TAT (78). Moreover, the isomeric γ emissions with energy suitable for SPECT imaging grants ^225^Ac the theranostic possibility (68, 69). Although the feasibility of using ^225^Ac for imaging is debatable because the amount administered for therapy may not produce enough gamma emission to be effectively detected by gamma camera.

The initial focus for harnessing the therapeutic potential of ^225^Ac was to identify a suitable chelating agent for *in vivo* delivery of ^225^Ac to target cells (79, 80). DOTA remains the gold standard for ^225^Ac labeling for all clinical work. Examples include ^225^Ac-PSMA-617, ^225^Ac-DOTATOC, ^225^Ac-DOTATATE, and ^225^Ac-DOTA-HuM195 (81). The first-in-human phase I dose escalation trial used ^225^Ac-DOTA-HuM195, which not only demonstrated the safety of ^225^Ac and its antileukemic activity, but also suggested that targeted therapy with an *in vivo* α-particle nanogenerator is a feasible approach in humans (82). With this proof of concept, ^225^Ac was then attached to PSMA-617 for prostate cancer therapy (83–86) and also bound to SSA-based pharmaceuticals for treating NETs.

## Preclinical studies of actinium-225-peptide receptor radionuclide therapy

Several preclinical studies tested the efficacy of α-particle emitting conjugates ^225^Ac-DOTATATE and ^225^Ac-DOTATOC in xenografted NET models and tested their toxicity as well as the biological effects of ^225^Ac. γH2AX was suggested as early key parameter in predicting tumor response to α-PRRT. The great reduction of growth and improved efficacy compared with ^177^Lu-labeled SSAs suggested that ^225^Ac-DOTATATE/DOTATOC has significant potential for improving PRRT in NETs and for the clinical translation in NET.

## Clinical application of actinium-225-peptide receptor radionuclide therapy

The first clinical study of ^225^Ac-PRRT in NET treatment was started in 2011 as a collaboration between the Joint Research Centre in Karlsruhe (Germany) and the University Hospital Heidelberg to treat patients with progressive NETs using ^225^Ac-DOTATOC. Based on 46 treatment cycles in 34 patients, the maximum tolerable dose was determined to be 40 MBq. The treatment was found to be safe with doses of 18.5 MBq every 2 months or 25 MBq every 4 months, and a cumulative activity of 75 MBq in regard to delayed toxicity. Despite the treatment response observed in several patients, further investigations were found to be necessary to improve patient selection and dosage regimens (87). Since then, clinical studies of ^225^Ac-PRRT in different NETs focused on whole-body SPECT/CT imaging possibility, efficacy and safety, therapeutic effect, as well as comparison to β-PRRT, has burgeoned (Table 3).

**TABLE 3:** Actinium-225-peptide receptor radionuclide therapy in NET treatment.

Peptide	Tumor types	Model	Key findings	Authors	References
**Pre-clinical**					
DOTATOC	Pancreatic NET	Xenograft	Activity up to 20 kBq had no significant toxic effect. Effective accumulation in xenografted NETs Reduced growth of NETs, and improved therapeutic efficacy	Miederer et al.	(117)
DOTATOC	Pancreatic tumor cells	*In vitro*	^225^Ac and ^177^Lu triggered-γH2AX-foci formation is an early key parameter in predicting response to internal radiotherapy.	Graf et al.	(156)
DOTATATE	Lung NET	Xenograft	1st Preclinical study for ^225^Ac-DOTATATE; Activity up to 111 kBq had no significant toxicity Significantly decreased tumor volume, increased tumor growth delay, and prolonged time to experimental endpoint for animals bearing both tumor types	Tafreshi et al.	(157)

**Peptide**	**Tumor types**	**Patient number**	**Key findings**	**Authors**	**References**

**Clinical**					
DOTATOC	NETs	34 patients	Promising treatment efficacy in various patients Suggesting comparative trials of α and β are needed.	Kratochwil et al.	(87)
DOTATOC	NETs	10 patients	The very first intra-arterial targeted alpha peptide radionuclide therapy using ^225^Ac DOTATOC ^225^Ac DOTATOC PRRT was very well-tolerated and effective.	Zhang et al.	(158)
DOTATATE	GEP-NET	32 patients	First clinical experience on efficacy and safety ^225^Ac-DOTATATE TAT as a promising treatment option for patients who are refractory to ^177^Lu-DOTATATE therapy	Ballal et al.	(159)
DOTATATE	Gastric NET	Case report	First whole-body and SPECT/CT images demonstrating high tumor uptake of ^225^Ac -DOTATATE.	Ocak et al.	(160)
DOTATATE	Rectal NET	Case report	Whole-body and SPECT/CT imaging results encourage the use of ^225^Ac -DOTATATE as a primary modality of treatment in advanced NET with metastases.	Kamaleshwaran et al.	(161)
DOTATOC	Liver NET	Case report	^225^Ac-PRRT in a β-radiation-refractory NET patient was shown to be safe and effective.	Zhang et al.	(162)
DOTATOC	Thymus NET	Case report	No adverse effects observed after ^225^Ac-DOTATOC TAT in patients with metastatic neuroendocrine tumors failing β-PRRT.	Zhang et al.	(163)
DOTATATE	Rectal NET	Case report	Using ^225^Ac-DOTATATE as first-line treatment presents a novel strategy for metastatic NETs with high skeletal disease burden.	Satapathy et al.	(164)
DOTATATE	NET-CUP	Case report	First case report demonstrating thyroid dysfunction developed after ^225^Ac-DOTATATE therapy in a patient with NET with unknown primary.	Kavanal et al.	(165)
DOTATATE	Pancreatic NET	Case report	^225^Ac-DOTATATE was well-tolerated at early stage of treatment, and patient demonstrated excellent response.	Budlewski et al.	(166)
DOTATATE	NET-CUP	Case report	First case who received actinium-225 first line with almost complete response at a single dosage	Alan Selçuk et al.	(167)
DOTATATE	GEP-NET	91	^225^Ac-DOTATATE TAT showed improved overall survival, even in patients refractory to prior ^177^Lu-DOTATATE treatment with transient and acceptable adverse effects	Ballal et al.	(168)

Considering the minimal/acceptable side effects, and the improved therapeutic efficacy and survival, one can conclude that ^225^Ac-PRRT not only provides an alternative in the treatment of β-radiation-refractory NETs, but also presents as possible frontline in treatment of NETs and can potentially usher a new era in radiopharmaceuticals even in tumors beyond NET.

## Challenges of using actinium-225 in peptide receptor radionuclide therapy

### Production of actinium-225

Limited ^225^Ac supply poses the most important challenge for widely implementing ^225^Ac-based PRRT. For more than two decades, the radiochemical extraction from ^229^Th, which is originated from the decay of the fissile isotope ^233^U (Figure 4), has been the most utilized strategy for the production of ^225^Ac and its daughter ^213^Bi (88, 89). Even today, ^225^Ac used in all clinical and virtually all preclinical studies is still obtained from the decay of ^229^Th. Worldwide, only three sources of ^229^Th are available (81). The Directorate for Nuclear Safety and Security of the Joint Research Centre (JRC) of the European Commission in Karlsruhe, Germany, the first laboratory to prepare ^225^Ac/^213^Bi, has produced approximately 13 GBq ^225^Ac annually since the 1990s for their center and a wide network of clinical collaborators (89, 90). The Oak Ridge National Laboratory (ORNL), USA produces up to 33 GBq per year for extensive application of treatment (91), while the Institute of Physics and Power Engineering (IPPE) in Russia reported an estimated production of 22 GBq annually (92) with no direct clinical application reported yet to our knowledge. This amounts to a current global production of approximately 68 GBq per year. At this level of supply, ^225^Ac-based treatments will continue to only be available for some few 100 patients per year, which obviously is insufficient to meet the growing demand of ^225^Ac labeled compounds in hospitals worldwide (81, 93).

**FIGURE 5:**
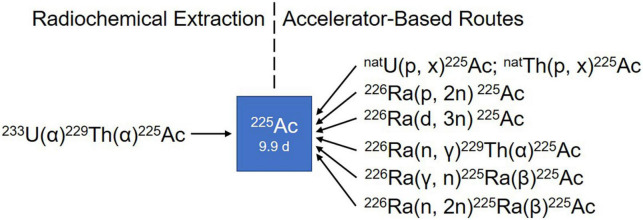
Production routes of ^225^Ac.

Consequently, multiple accelerator-based routes to scale up ^225^Ac production have been investigated, including the irradiation of ^226^Ra target using protons, deuterons or gamma-rays and the spallation of ^nat^Th or ^nat^U targets with highly energetic protons (Figure 5) (94). Among the routes described so far, the spallation of ^nat^Th is currently the most frequently used accelerator-based route. Even though the feasibility of the process has been demonstrated in the USA (95, 96) and Russia (97, 98), the implication of coproducing the long-lived ^227^Ac (*t*_1/2_ = 21.8 years) as impurity is a serious limitation in terms of clinical translation and waste management (99). In addition, issues with licensing and clinical safe handling need to be resolved (81).

Medium-energy proton irradiation of ^226^Ra in a cyclotron using the reaction ^226^Ra(p,2n)^225^Ac offers a number of advantages over the ^nat^Th spallation, and is currently the most promising method of large-scale and cost-effective production of ^225^Ac. Chemical purification of the irradiated targets generates ^225^Ac with high isotopic purity because there are no other long-lived actinium isotopes, such as ^227^Ac, co-produced (100). It is important to mention that the availability of appropriate cyclotrons worldwide [energy range 15–25 MeV (101)] makes the production of ^225^Ac feasible for basic and applied research (94). The downsides of this approach are related to the preparation and safe handling of targets containing milligram of radioactive ^226^Ra and managing its highly radiotoxic gaseous decay product ^222^Rn. Further research on the practical implementation of this production route is required to meet the high demand in the mid-term future (81).

The reaction ^226^Ra(d,3n)^225^Ac has been suggested as an improved approach for producing ^225^Ac (102). Model calculations are predicting an increased production yield compared to the ^226^Ra(p,2n)^225^Ac reaction. However, deuteron irradiation leads to an enhanced co-production of ^226^Ac *via* the ^226^Ra(d,2n)^226^Ac reaction, resulting in an extended cooling time necessary to allow for ^226^Ac decay. Moreover, limited accelerators are available worldwide that can provide deuteron beams of sufficient energy and the complicated handling of ^226^Ra and its decay product remains an issue (81).

Other production routes being studied involve ^226^Ra(n, γ)^229^Th(α)^225^Ac, ^226^Ra(γ,n)^225^Ra(β)^225^Ac, and ^226^Ra(n,2n)^225^Ra(β)^225^Ac. It is important to point out that the main limitation of all these strategies is the handling of radium target and the generation of long-lived co-products, such as ^227^Ac (*t*_1/2_ = 21.77 years) and ^228^Ac (*t*_1/2_ = 1.9 years). However, preliminary results demonstrated by these above-mentioned methods are promising (94, 100, 103–105). Unfortunately, but understandably, these supply limitations bring about a high cost that is considered unaffordable by many researchers.

### Imaging

Clinical imaging using ^225^Ac also presents challenges. Therefore, post-therapy imaging is usually not done after ^225^Ac administration for tracer localization. The use of two photopeaks at 218 keV and 440 keV has long been suggested for clinical imaging of α-particles (106), until recently, Rasheed et al. focusing on gamma-ray spectrum for ^225^Ac showed an additional third photopeak at 78 keV, with higher counting density (107). Although imaging using the additional 78 keV photopeak was suggested to yield higher counts, better images, and more lesion delineations, the literature showing the feasibility of using the three photopeaks is limited to only few clinical case reports (108, 109).

### Dosimetry

The short mean free path of ^225^Ac (110), as well as the complexity and timing of ^225^Ac decay in relation to its radiopharmaceutical stability, uptake and clearance makes measurement of ^225^Ac activity very difficult. A number of preclinical studies have estimated ^225^Ac activity using measurements related to γ-emissions (111). However, a low probability of γ-emission and overlapping Bremsstrahlung due to β-emitters in the ^225^Ac decay chain preclude simultaneous treatment and dosimetry measurement in a clinical setting (84). Thus, current clinical TAT research relies greatly on indirect approximation by extrapolating pre-existing ^177^Lu-labeled pharmaceuticals (84, 112, 113). Preclinical studies focusing on ^225^Ac have used the standard approach described by the Medical Internal Radiation Dose (MIRD) committee. Total dosimetry was calculated from the summation of doses of ^225^Ac, ^221^Fr, ^217^At, ^213^Bi, and ^213^Po recorded in a biodistribution study (111). Unfortunately, collection of biodistribution data for dosimetry estimation is not possible for most α-emitters with therapeutic potential. This makes a direct and accurate preclinical dosimetry measurement for ^225^Ac to be extrapolated into and to guide clinical trials, as well as standardizing α-dosimetry measurement highly demanded in clinical application.

### Chelator

Finding a chelator to accommodate ^225^Ac and its progenies with sufficient stability was proven a great challenge given the range of different periodic properties of the daughters of ^225^Ac. The recoil effect associated with α-decay of ^225^Ac imparts an energy that is thousands of times greater than the binding energy of any chemical bond (75), resulting in an inevitable release of the daughter nuclide from the chelate moiety. Subsequently, the unbound α-emitting daughter nuclides are redistributed *in vivo* causing substantial harm to normal tissue and reducing the therapeutic effect. For example, the renal toxicity induced by ^213^Bi (in small animals) is considered a critical constraint to clinical use of ^225^Ac (114). However, recent results indicated that the release of free metals from DOTA may be not as evident as expected shown by fractioned radio-HPLC (115).

Finding a new chelator is one of the strategies to improve ^225^Ac-TAT. McDevitt et al. presented a comparison of ^225^Ac radiolabeling efficiency and *in vitro* stability of multiple chelators including DTPA, DOTA, TETA, DOTPA, TETPA, and DOTMP (116), and concluded that only DOTA and DOTMP showed chelation of ^225^Ac after 2 h at 37°C with radiochemical yields of >99 and 78%, respectively. Further *in vitro* serum stability testing showed that the ^225^Ac-DOTA complex outperformed the rest with >90% of complexes still intact after 10 days (104). Due to its outstanding stability, DOTA remains the gold standard for ^225^Ac-radiolabeling for all clinical research. However, DOTA has a decreased thermodynamic stability when used with larger metal ions. Moreover, chelation of ^225^Ac is a slow reaction demanding extensive heating and high amount of ligand for an adequate yield (77, 116, 117). Macropa, crown, and py4pa are new chelators with improved radiochemical yield and specific activity, as well as achievable labeling conditions. However, none of the new chelators was investigated enough so far to confirm *in vivo* stability, and their potential to translate into clinical application is yet to be assessed (118–122).

### Quality control of actinium-225-radiopharmaceuticals

In addition to complicated handling, standardized quality control of radiopharmaceuticals remains a problem in routine clinical production. Because of the complicated decay chain of Ac-225 (Figure 4), special methods must be used for both measurements of the activities and for quality control by radio-thin-layer chromatography (radio-TLC) and radio-high-performance-liquid chromatography (radio-HPLC). Since alpha emitters are difficult to detect directly, indirect measurements are usually used for all measurements by detecting the daughters Fr-221 or Bi-213 (gamma emitters). Fr-221, with its short half-life of 4.9 min, is nearly in radioactive equilibrium after 60–120 min (123). In contrast, Bi-213, with a half-life of 45.6 min, takes hours to reach equilibrium. Thus, measurements of Fr-221 are often used for a faster quality control and release within a clinical environment. When separating free metals and labeled compounds on a TLC plate, the plate is equilibrated for an hour after development before being measured on a TLC scanner. During this period, a constant amount of Fr-221 has formed for both possible species, so comparison is possible, and a labeling yield can be determined. Afterward, an additional distinction must be made between the activity caused by the decay of Fr-221 and the activity of Bi-213. This can typically be differentiated in a gamma spectrometer by splitting the plate. Fr-221 has a line at 218 keV and Bi-213 at 440 keV (122, 124). However, the measurement with radio-TLC is not a valid method to determine the yield and purity of a radiopharmaceutical and additional radio-HPLC methods are required. Two possibilities are conceivable here. Direct detection of Ac-225 by liquid scintillation detection is relatively easy to implement, but may require large activities for injection, which is not always practical and can be very expensive. Therefore, indirect detection of the daughters can be used again by the fractionated collection of the measured sample and subsequent analysis of their components in a gamma spectrometer. In this way, the retention behavior of the compounds on the column can be investigated even when injecting small quantities, and possibly more precise statements can be made about side- or decomposition-products (115).

## Future of actinium-225-peptide receptor radionuclide therapy

### Combination with somatostatin receptor antagonist

Today, the development of novel SSTR antagonists holds promise for enhanced diagnostic accuracy and efficacy of SSTR-mediated imaging and therapy. SSTR2-selective ^111^In-DOTA-BASS (125) and SSTR3-selective ODN-8 (126, 127), the first generation of radiolabeled SSTR antagonists all recognized a larger number of binding sites and revealed an in general twofold higher uptake level *in vitro* than their agonist counterparts (128). Despite this, BASS labeled with ^64^Cu *via* the chelator 4, 11-bis(carboxymethyl)-1,4,8,11-tetraazabicyclo [6.6.2]hexadecane (CB-TE2A) (^64^Cu-CB-TE2A-BASS) showed a compromised tumor uptake compared to the agonist ^64^Cu-CB-TE2A-octreotate in SSTR2-positive AR42J xenografts (129).

This suboptimal tumor uptake triggered the second generation of radiolabeled SSTR antagonists. In 2006, Ginj et al. presented the idea that radiolabeled SSTR antagonists may perform better than agonists despite the lack of receptor-mediated internalization (127). Despite its lack of internalization, SSTR antagonist demonstrated higher tumor uptake with a higher tumor-to-normal ratio and longer tumor retention time than that of agonist, possibly due to its capability to bind larger variety of receptor conformations, than that of SSTR agonists (130). The initial evidence that antagonists are superior to agonists guided the design and development of more potent SSTR2 antagonists with improved affinity (127, 131), with some of the potential antagonists studied as radiotracers. Among all the potential SSTR2 antagonists, LM3 and JR11 were the most interesting, having the highest hydrophilicity and best affinity. These two were evaluated in a comprehensive study, in combination with two chelators DOTA and NODAGA, and multiple radiometals (132, 133). Interestingly, ^68^Ga-DOTA-JR11 and -LM3, which have drastically lower affinities for SSTR2 (approximately 150-fold and 60-fold, respectively) than ^68^Ga-DOTATATE, showed higher tumor uptake (132). Likewise, the therapeutic counterpart ^177^Lu-DOTA-JR11 exhibited a higher tumor uptake, a longer tumor retention time, and an improved tumor-to-kidney ratio compared to ^177^Lu-DOTATATE (134), and hence led to a delayed tumor growth and an extended median survival period (135).

Lutetium-177-DOTA-JR11 was first compared in a pilot study with ^177^Lu-DOTATATE. In the same four patients with grade 1–3 metastatic NET, it showed a 1.7–10.6 times higher tumor dose, and at the same time a 1.1–7.2 times higher tumor-to-kidney and tumor-to-bone marrow ratio, compared with ^177^Lu-DOTATATE (136, 137). A phase I study with 20 grade 1–3 patients reported a best overall response of 45% (RECIST 1.1 criteria) and a median progression-free survival of 21 months (95% CI: 13.6-not reported). Unfortunately, grade 4 hematotoxicity was reported in 4 out of 7 patients after two cycles of ^177^Lu-DOTA-JR11, resulting in the suspension of this therapy protocol (138). The protocol was under subsequent modification to limit cumulative absorbed bone marrow dose. A phase I/II open-label study (Clinical trial identification: EudraCT: 2015-002867-41; NCT02592707) is currently ongoing to evaluate the safety and efficacy with the adjusted treatment regimen. Hitherto, only one abstract summarizing the promising efficacy and low toxicity is available. In 20 patients with adequate follow-up, no grade 3/4 renal toxicities and a 90% (95% CI: 68.3–98.8%) disease control rate was reported (139).

In parallel to ^177^Lu-DOTA-JR11, ^177^Lu-DOTA-LM3 was evaluated in 51 patients with metastatic neuroendocrine neoplasm at the Theranostics Center for Molecular Radiotherapy and Precision Oncology in Wiesbaden, Germany. ^177^Lu-DOTA-LM3 was reported to have a 3–5.1 times higher absorbed doses and a 22 h longer whole-body effective half-life than the agonist ^177^Lu-DOTATOC. All patients tolerated therapy well without any serious acute adverse effects, in particular, there was no nephrotoxicity observed (130).

**FIGURE 6:**
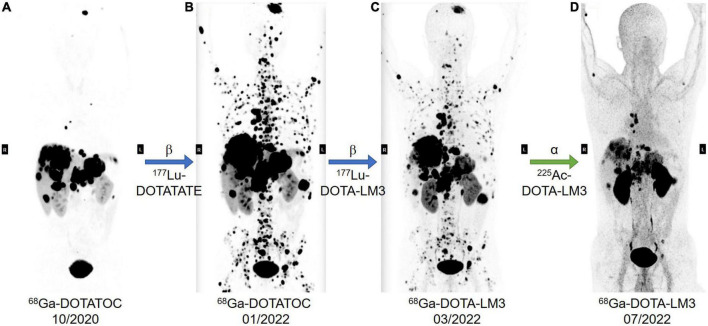
A 40-year-old patient was diagnosed with poorly differentiated non-functioning pancreatic-NET with bilobar liver and extensive bone metastases, Ki-67 index of 25% NEN-G3 without known mutations. The patients had previously undergone laparoscopic subtotal pancreatic resection and splenectomy, CAPTEM chemotherapy, transarterial chemoembolization (TACE) of right liver lobe and resection of abdominal lesion. In addition, the patient had previous ineffective treatment with Lanreotide, Everolimus, and Sunitinib. In total, the patient had received eight cycles of PRRT, and had a very poor prognosis, extensive bone metastases, even after multiple cycles treatment with ^177^Lu-DOTATATE (**A,B**, ^68^Ga-DOTATOC PET/CT before and after ^177^Lu-DOTATATE treatment). The 9th PRRT cycle was performed with ^177^Lu-DOTA-LM3 (**B,C**, PET/CT imaging before and after ^177^Lu-DOTA-LM3 treatment), and the last two cycles with ^225^Ac DOTA-LM3 (**C,D**, PET/CT imaging before and after ^225^Ac-DOTA-LM3 treatment), 10 MBq in March and 15 MBq in May 2022, respectively. The cumulative administered radio activities are 66.7 GBq of ^177^Lu and 25 MBq ^225^Ac. Restaging result in July 2022 showed excellent response to ^225^Ac-DOTA-LM3 treatment with partial remission according to the THERCIST. As shown in the most recent PET/CT, there is dramatic improvement, especially concerning the previous innumerable bone metastases in the spine, ribs, and pelvis. The primary tumor in the pancreas, and the liver and bone metastases further decreased in size and number, and no new metastatic lesions were noted. The patient felt dramatically better in comparison to the previous treatments. After the last PRRT, he only experienced mild alopecia and mild pain in the upper right abdomen over 1 week, and has been physically active and gained 3 kg body weight over the past 2 months.

Here we would like to showcase our recent therapeutic result of PRRT using ^225^Ac-DOTA LM3 to demonstrate the exciting potential of ^225^Ac labeled SSTR antagonist in NET treatment (Figure 6). A 40-year-old pancreatic-NET patient suffered from bilobar liver metastases and extensive bone metastasis even after multiple cycles of PRRT with ^177^Lu-DOTATATE, and compromised therapeutic response after one cycle of ^177^Lu-DOTA-LM3 treatment, was suggested for PRRT with ^225^Ac-DOTA-LM3. This case with β-radiation refractory metastatic NET demonstrated incredibly auspicious therapeutic response after two cycles of ^225^Ac-DOTA-LM3, especially concerning the bone metastases. With this successful case reported for the first time, we would like to suggest that PRRT with ^225^Ac-labeled SSRT antagonist can potentially be a game changer in therapeutic nuclear medicine, and a promising cure for metastasized tumor.

Moreover, the pharmacodynamics of SSTR antagonists have been studied as the clinical interest for them is rapidly growing. The antagonist showed faster association, but slower dissociation, as well as longer cellular retention time compared to the agonist. Moreover, antagonists recognize more binding sites than agonists, providing more targeting opportunities (140). Taking the proposed mechanism, preclinical and clinical studies into consideration, it is obvious that using radiolabeled SSTR antagonists, especially LM3 and JR11, may provide more successful imaging and PRRT strategies for neuroendocrine tumors, even those with relatively low SSTR expression (130, 137, 141) compared to agonists.

### Cocktail approach

Most PRRTs using radiolabeled SSAs and antagonists has until now depended substantially on the most prominently expressed SSTR2 (142). However, NET expression of SSTR subtypes is heterogeneous, and things are made even more difficult by contradictory expression profiles due to different detection methods (20, 143–153). Also, the downregulation or loss of SSTR2 in advanced stages is inherently associated with worse disease prognosis, compromised image sensitivity, and suboptimal therapy with SSTR2-specific radiolabeled SSAs. Using SSTR agonists and antagonists with affinity to more SSTR subtypes is therefore a proposed method—a cocktail approach—of great clinical interest (154). An impressive clinical case showing the application of this cocktail approach, where six tracers were given to a single patient with prostate cancer, was reported at the 2016 SNMMI Highlights Lecture (155). Inspired by the cocktail approach in treating prostate cancer, using SSTR analogs and antagonists targeting various SSTR subtypes in combination with ^225^Ac-PRRT is worth exploring.

## Conclusion

Peptide receptor radionuclide therapy in NET patients has come a long way since Krenning’s first treatments in the 1990s. Novel radionuclides are constantly being developed and tested, in a race to find the perfect theranostic pair. Modified chelators and new ligands including SSTR antagonists are gaining more and more attention, the latter in particular as they have been revealed to give great tumor uptake, retention time and tumor-to-background ratio. ^225^Ac in particular is very worth investigating.

The rise of the α-emitters as a compliment or replacement to β-emitters is one of the most exciting recent developments. The shorter range gives “precision” in precision medicine a new meaning: Even less damage to surrounding healthy tissue and even more powerful damage to tumor cells.

To this date, confirmed literature on PRRT using an α-emitter with a SSTR antagonist has yet to be published, and obviously, there are still many hurdles to overcome. Technical ones, such as how to best combine chemical moieties into a stable, pharmacokinetically feasible drug; economical ones, such as how to best implement a global mass production of radionuclides for research and clinical use; clinical ones, such as how to set a dosage of the existing theranostic pairs that minimizes toxicity whilst maximizing tumor uptake. Yet, to have the recent promising clinical studies on ^177^Lu-DOTA-LM3 and -JR11 in mind at the same time as the potentials of TAT is intriguing; hopefully a new and promising era for NET therapy will see daylight in the foreseeable future.

## Author contributions

JZ, XC, and RB: study conception and design. MS, VJ, and RB: data collection. MS and JZ: analysis and interpretation of results. MS and VJ: writing—original draft preparation. JZ, LG, P-LK, RB, and XC: writing—review and editing. All authors reviewed the results and approved the final version of the manuscript.
